# Preoperative Vitamin D Levels as a Predictor of Transient Hypocalcemia and Hypoparathyroidism After Parathyroidectomy

**DOI:** 10.1038/s41598-020-66889-8

**Published:** 2020-06-18

**Authors:** Ilknur Ozturk Unsal, Murat Calapkulu, Muhammed Erkam Sencar, Sema Hepsen, Davut Sakiz, Mustafa Ozbek, Erman Cakal

**Affiliations:** 1 Department of Endocrinology and Metabolism, University of Health Sciences, Diskapi Yildirim Beyazit Training and Research Hospital, Ankara, Turkey; 2 Department of Endocrinology and Metabolism, Mardin State Hospital, Mardin, Turkey

**Keywords:** Endocrinology, Medical research

## Abstract

Hypocalcemia is a common problem after parathyroidectomy and/or thyroidectomy. The complication may be transient or permanent. Most cases occur as a result of removal of the parathyroid glands or damage to the glands during neck surgery. The purpose of this study was to evaluate the effect of preoperative vitamin D deficiency in predicting transient hypocalcemia and hypoparathyroidism after parathyroidectomy.Retrospective evaluation was made of 180 patients with primary hyperparathyroidism in respect of serum 25(OH)D, calcium and parathyroid hormone before and after parathyroidectomy. Transient hypocalcemia was defined as corrected calcium ≤ 8.4 mg/dL, and these cases were then evaluated for preoperative 25(OH)D values. Transient hypoparathyroidism has been described as low PTH level immediately after surgery before beginning any supplementation. Permanent hypoparathyroidism is accepted as the need for medical treatment is necessary over 12 months.Both transient hypocalcemia and hypoparathyroidism developed at statistically significantly higher rates in patients with preoperative vitamin D deficiency and vitamin D insufficiency.Vitamin D deficiency is an independent contributor to transient hypocalcemia and hypoparathyroidism following parathyroidectomy.

## Introduction

Primary hyperparathyroidism (PHPT) is a result of the autonomous production of parathyroid hormone (PTH) from one or more abnormal parathyroid glands. PHPT is diagnosed in the presence of hypercalcemia and elevated or inappropriately normal (nonsuppressed) parathyroid hormone levels. However a small percentage of patients present with normocalcemia^1^. PHPT is more common in patients more than the aged >50–65 years, but can occur at any age, including in childhood. Prevalence is thought to be 1–7 cases per 1000 adults. The disease is more common in females and the ratio of females to males is 2–3/1^2^. PHPT is caused by a single parathyroid adenoma in approximately 85% of patients. In 15% of patients, it can be associated with hyperplasia but parathyroid carcinoma is a rare cause^3^. The signs and symptoms are a combination of the effects of increased PTH secretion as well as hypercalcemia. Both bone disease and nephrolithiasis are directly due to increased PTH levels, whereas anorexia, constipation, polydipsia, polyuria, and nausea are related more to hypercalcemia^2^. Hypercalciuria is a risk factor for nephrolithiasis, which is a complication of primary hyperparathyroidism. Kidney stones are present in approximately 7% of patients and are seen at increased compaired with the general population^4^. PHPT is a very common disease, especially in postmenopausal women. Moreover, bone turnover is increased with loss of cortical bone in PHPT^5^. In addition the risk of vertebral and nonvertebral facture appears to be increased, as suggested by data from epidemiological and cohort studies^6,7^. Epidemiological studies have suggested that there is a cardiovascular (CV) risk in elderly men, even within the normal PTH range. PHPT has also been associated with a state of insulin resistance and PTH is directly positively correlated to the Left Ventricular Mass Index in PHPT^5^.

Vitamin D3 is made in the skin from 7-dehydrocholesterol under the influence of UV light. Vitamin D is metabolized first to 25 hydroxyvitamin D (25OHD), then to 1,25-dihydroxyvitamin D (1,25(OH)2D). 1,25(OH)2 vitamin D, the active metabolite of vitamin D, also known as calcitriol, regulates not only calcium and phosphate homeostasis but also cell proliferation and differentiation, and has a key a role to play in the responses of the immune and nervous systems. Also, *in vivo* novel pathways of vitamin D3 metabolism were defined generating D3-hydroxy derivatives different from 25-hydroxyvitamin D3 [25(OH)D3] and 1,25(OH)(2)D3 in placenta, adrenal gland, and epidermal keratinocytes^8–11^.

Surgery is always an appropriate option for individuals with PHPT and operative management is more effective and less costly than either long-term observation or medical treatment. After a parathyroidectomy, nephrolithiasis incidence decreases, the bone mass densities of the lumbar spine and femoral neck increase compared to preoperative values, and fractures frequency decrease^12^.

Hypocalcemia is a common problem after parathyroidectomy and/or thyroidectomy. Gambardella *et al*. reported transient hypoparathroidism rates of 11.4% in total thyroidectomy vs 21.4% in total thyroidectomy with prophylactic central neck dissection and permanent hypoparathyroidism rates of 1.5% in total thyroidectomy vs 6.4% in total thyroidectomy with prophylactic central neck dissection^13^.

There are no specific data for the prediction and management of hypocalcemia in patients with parathyroidectomy. The hypothesis of the study was that preoperative vitamin D deficiency indicates higher risk for postoperative transient hypoparathyroidism and hypocalcemia in patients following parathyroidectomy.

## Materials and methods

### Study population

**Figure 1:**
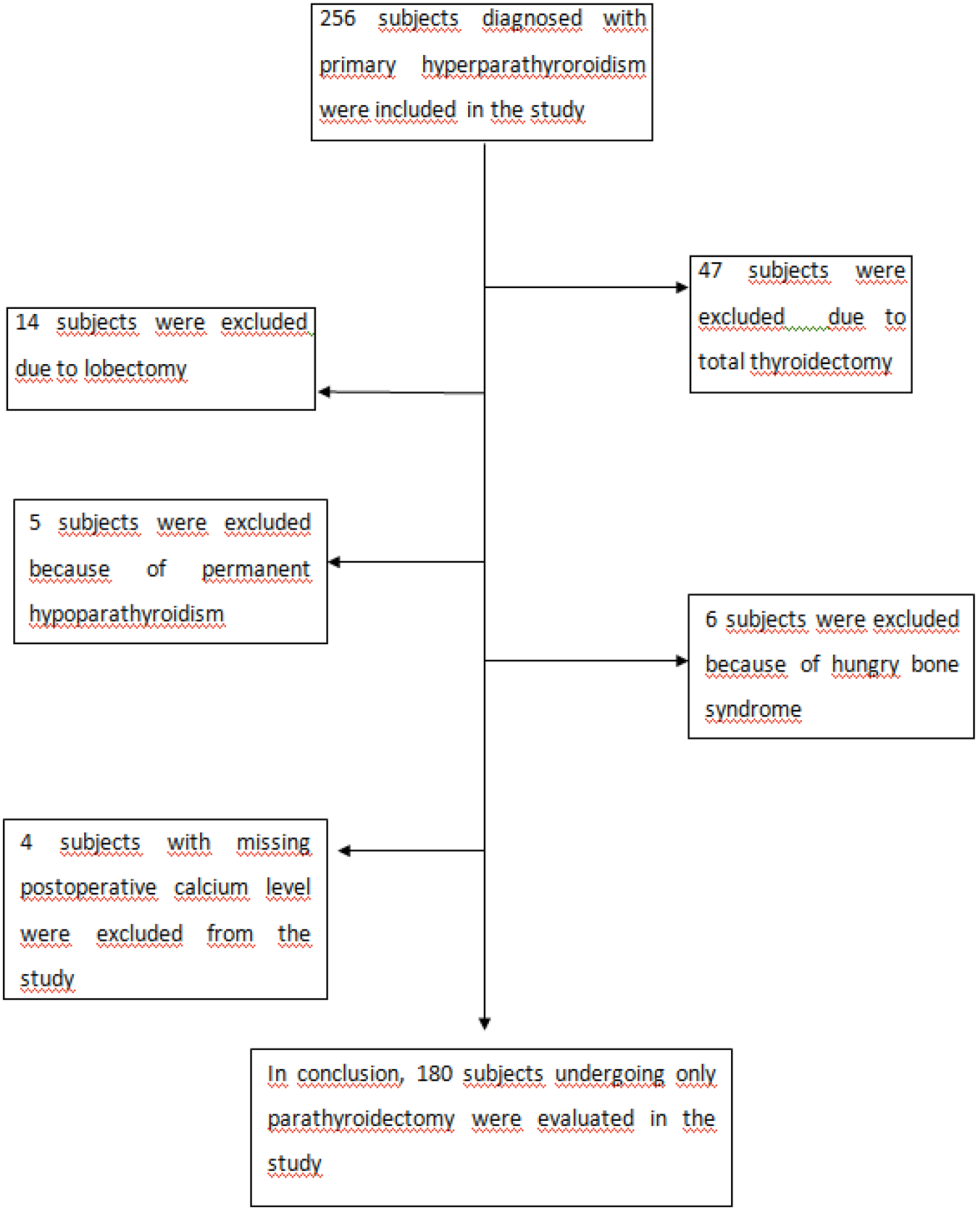
Study flow diagram.

This retrospective study included a total of 180 patients with primary hyperparathyroidism. Patients who underwent concomitant thyroidectomy, permanent hypoparathyroidism or were aged <18 years were not included in the study (Fig. 1). The study was performed at the Endocrinology Department of Diskapi Yildirim Beyazit Training and Research Hospital between November 2014 and December 2018. The protocol was approved by the Ethics Committee of University of Health Sciences, Diskapi Yildirim Beyazit Training and Research Hospital. Te study was conducted in accordance with the Declaration of Helsinki and all participants provided written informed consent before the study procedures. Demographic data were collected together with clinical and surgical details, postoperative laboratory values (until six months), and comorbidities. After parathyroidectomy, patients were evaluated for levels of PTH and serum calcium at the 24^th^–96^th^ hour. Transient hypocalcemia was accepted as serum albumin-corrected calcium level ≤8.4 mg/dL until thepostoperative fourth day. Vitamin D deficiency is defined as 25(OH)D ≤ 20 ng/mL, and vitamin D insufficiency as 25(OH)D of 20–30 ng/mL. Transient hypoparathyroidism was accepted as PTH ≤ 15 pg/mL.

### Clinical and biochemical evaluation

Serum Ca levels were analyzed using a Beckman Coulter Olympus AU5800 Clinical Chemistry autoanalyzer (Beckman Coulter Inc., Brea, California, USA). Serum total 25(OH)D and PTH were measured using a Beckman Coulter UniCel DxI 800 immunoassay systems autoanalyzer (Beckman Coulter Inc., Brea, California, USA). Reference ranges were described as calcium: 8.5–10.4 mg/dl, phosphorus: 2,5–4,5 mg/dL PTH: 15–74 pg/mL, alkaline phosphatase (ALP): 30–120 U/L, and 24-hour urine calcium: 100–321 mg/day.

### Statistical analyses

Statistical analyses were performed using SPSS software (version 23.0, SPSS, IBM Corporation, NY, USA). The Kolmogorov-Smirnov test was used to assess the conformity of the data to normal distribution. Categorical data were presented with frequencies and percentages (%). All continuous data with normal distribution were presented as mean ± standard deviation (SD), and in the case of non-normally distributed data were presented as median (range) values. The Kruskal-Wallis test was performed to compare non-normally distributed data. The relationships between categorical variables were examined using Chi-square analysis. A value of p < 0.05 was considered statistically significant.

## Results

**Figure 2:**
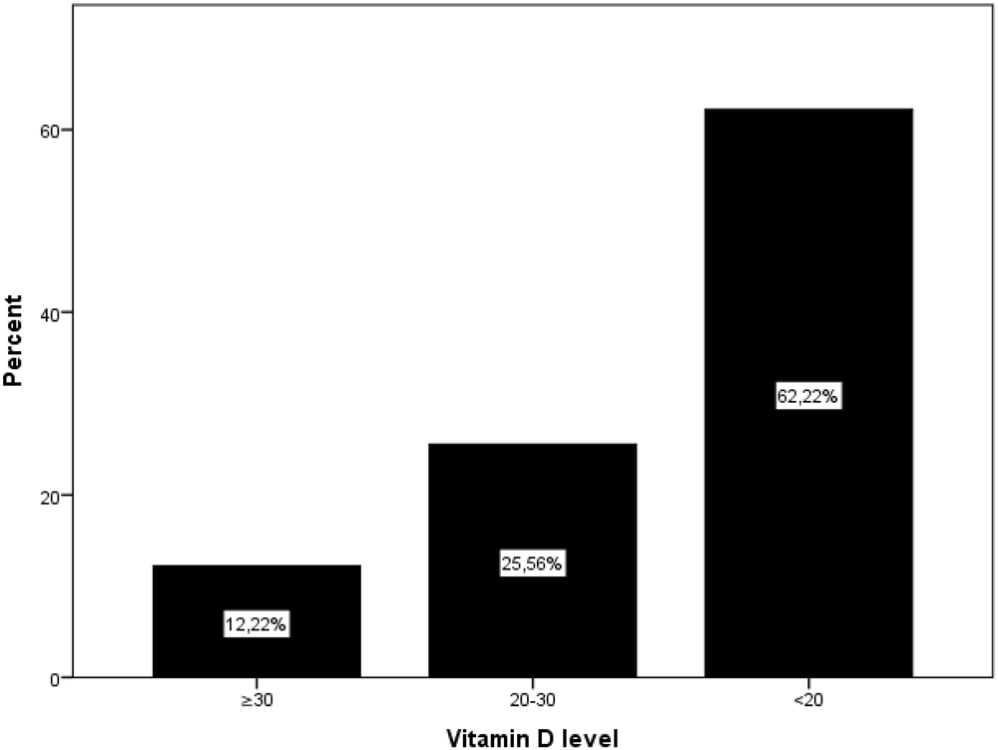
Preoperative vitamin D status.

**Figure 3:**
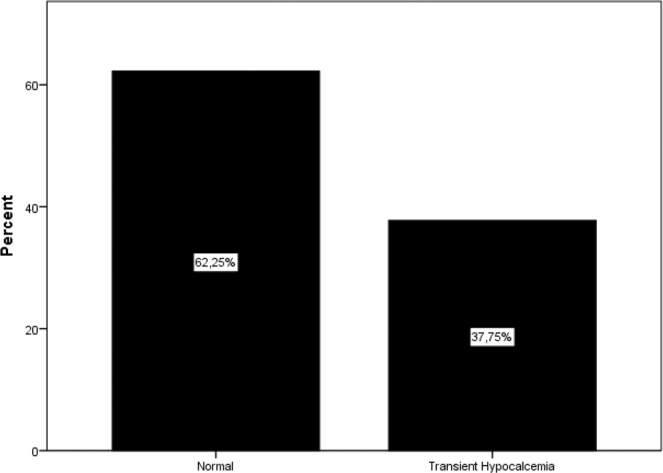
Transient hypocalcemia.

**Figure 4:**
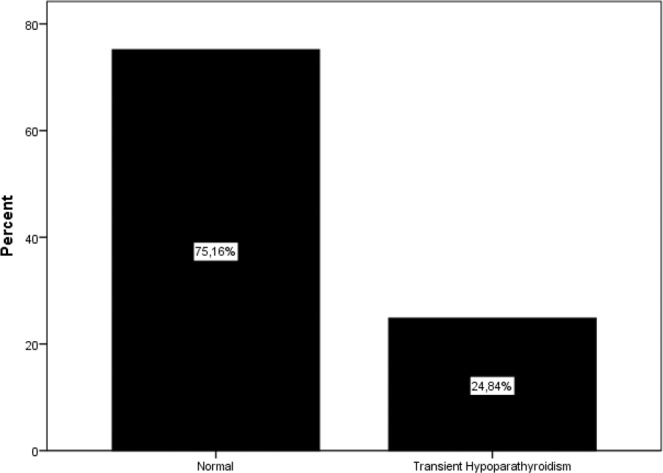
Transient hyoparathyroidism.

Initially, 256 patients with primary hyperparathyroidism were enrolled in the study, and after the exclusion of 76 patients for various reasons (Fig. 1), and the study was continued with 180 patients. Only patients who underwent minimally invasive parathyroidectomy or unilateral parathyroidectomy were included in the study. The mean age of patients was 54.9 ± 12.2 years, and the majority 147/180, (81.7%) were female. The demographic characteristics and biochemical parameters of the patients are shown in Table 1. Postoperative histopathological examination demonstrated adenoma in 156 (%87,3) cases, hyperplasia in 13 (%10,5) cases, carcinoma in 2 (%1.1) cases, and double adenoma in 2 cases (%1,1). Vitamin D deficiency was determined in 62.22% of patients and vitamin D insufficiency 25.56% of patients (Fig. 2). Preoperatively, 22 patients (12.2%) had normal levels of vitamin D. At the end of the study, 37% of patients were determined with transient hypocalcemia and 24% with transient hypoparathyroidism (Figs. 3 and 4). After the parathyroidectomy median calcium levels were low in both vitamin D deficiency and insufficiency groups compared with the normal D vitamin group (p = 0,02) (Table 2). The rate of transient hypocalcemia and transient hypoparathyroidism was statistically significantly higher in the groups with vitamin D deficiency or insufficiency compared to the normal vitamin D level group (p = 0.02, p = 0.04, respectively) (Table 3).

**Table 1:** Demographic and clinical data of primary hyperparathyroroidism patients before parathyroidectomy.

N	180
F/M	147 (81.7%)/33 (17.3%)
Age (year)	54.9 ± 12.2
TSH (0.38–5.33 mIU/L)	1.9 ± 1.1
Serum Calcium (8.5–10.4 mg/dL)	11.2 ± 0,8
PTH (15–74 pg/mL)	131.5 (61–922)
Serum Phosphorus (2.5–4.5 mg/dL)	2.6 ± 0,5
ALP (30–120 U/L)	107 (47–773)
24-hour urinary calcium level (100–321 mg/dL)	338 ± 172
25(OH)D (ng/mL)	18.1 ± 10.7

**Table 2:** Postoperative serum calcium, PTH, and phosphorus levels.

	Vitamin D level			
	≥30 ng/mL	20–30 ng/ml	<20 ng/ml	p value
Calcium (mg/dl)	9,45 (8.6–10,4)	8,97 (7,6–10,2)	8,9 (7,6–10,4)	0.02
PTH (pg/mL)	13,7 (5,7–89)	10,2 (0,3–105)	14 (0,3–111)	0.59
Phosphorus (mg/dL)	3,6 (2,5–4,1)	3,3 (1,9–4,1)	3 (1,56–4,8)	0.14

**Table 3:** Comparison among three vitamin D groups with chi-square analysis.

	Vitamin D level			
	≥30 ng/mL	20–29 ng/mL	<20 ng/mL	p
Transient Hypocalcemia (%)	11,1	33,3	44,3	0.02
Transient Hypoparathyroidism (%)	9,1	16,7	31,3	0.04

## Discussion

PHPT is a common endocrine disorder due to a surge in incidental diagnosis with routine laboratory testing. Surgery is always the most suitable option for individuals with PHPT, with focused exploration and bilateral parathyroid exploration as the standard surgical options for patients with primary hyperparathyroidism^14^. Commonly accepted indications for parathyroidectomy are osteoporosis, fragility fracture, nephrolithiasis, hypercalciuria, renal insufficiency, moderate hypercalcemia, and age <50 years^15^. Hypocalcemia is an important potential complication of parathyroid exploration. Hypocalcemia may be transient or permanent. Parathyroid autotransplantation can be used to reduce the risk of permanent hypocalcemia, although this does not affect transient hypocalcemia, because reimplanted glands achieve normal function after 3 to 14 weeks^16–18^. Transient hypoparathyroidism may be due to manipulation of the blood supply to or removal of one or more parathyroid glands during surgery, whereas permanent hypoparathyroidism is due to a decreased parathyroid reserve. The risk of postoperative hypoparathyroidism and hypocalcemia increases in particular with extensive thyroidectomy, completion procedures, and central neck dissection. Previous studies have shown that bilateral parathyroid exploration is associated with higher rates of postoperative hypocalcemia^14,19,20^. The current study included only patients who underwent minimally invasive parathyroidectomy or unilateral parathyroidectomy. In a previous meta-analysis of 12743 cases, postoperative hypocalcemia was determined at the rate of 1.6% in focused parathyroid exploration vs 13.2% in bilateral exploration^14^.

Philips *et al*. found PTH to be a significant predictor for hypocalcemia after unplanned parathyroidectomy. PTH ≤ 15.5 significantly increases the risk of developing hypocalcemia, and prophylactic ≥1000 mg elementary calcium is recommended for these patients^12^. In another study, the preoperative PTH level was found to be one of the most important factors associated with postoperative hypocalcemia in patients who underwent thyroidectomy^21^. Soares *et al*. showed that postoperative hypocalcemia was associated only with parathyroid hormone and the preoperative vitamin D levels of patients were no different in those with or without hypocalcemia. In that study serum 25(OH)D concentrations were not found to be predictors for hypocalcemia^22^. Falcone *et al*. concluded that 25(OH)D did not predict postoperative hypocalcemia^23^. Lang *et al*. reported that preoperative 25(OH)D deficit (≤20 ng/mL) did not increase the post-thyroidectomy hypocalcemia rate^24^. However, Al-Khatib *et al*. showed that patients with preoperative 25OHD levels ≤25 nmoL/L had a 7.3 fold higher risk of developing post-thyroidectomy hypocalcemia^25^. Hence, the role of vitamin D level as a predictor for hypocalcemia is still controversial.

Interestingly, in the current study, a relationship was observed between postparathyroidectomy transient hypocalcemia and preoperative 25(OH)D levels, and the 25(OH)D level was seen to predict hypocalcemia in patients with primary hyperparathroidism after parathyroidectomy. Theoretically, patients with reduced serum Vitamin D levels are more prone to develop hypocalcemia due to a higher dependency on PTH-induced bone and renal re-absorption mechanisms^22^. Decreased serum calcium due to functional hypoparathyroidism causes reductions in bone reabsorption and increases in bone formation and an increased influx of calcium into bone. Following parathyroidectomy in patients with primary hyperparathyroidism, hypocalcemia is further exacerbated by increased calcium excretion and decreased intestinal calcium absorption owing to reduced PTH-mediated renal 1,25 dihydroxyvitamin D production further exacerbate a hypocalcemia^12^. In patients with vitamin D deficiency, calcium homeostasis is provided by increasing PTH secretion in the blood. In the current study, transient hypoparathyroidism was determined more in the vitamin D deficiency and insufficiency groups (p = 0.04). This may be related to the higher preoperative PTH level than normal due to the secondary hyperparathyroidism effect. In a previous study, the rate of hypocalcemia was found to be high in patients with preoperative PTH near the upper limit (p < 0.01)^21^. Therefore, the preoperative PTH level in vitamin D deficiency may not reflect the reality.

The main limitation of this study was the retrospective design, and not measured urine calcium creatinine ratio. But we were collected 24 hour urine second time in patients who we do not sure 24 hour urine calcium excretion value. However, to the best of our knowledge, this is the first study in literature to have investigated the relationship between postoperative hypocalcemia and vitamin D after parathyroidectomy.

In conclusion, parathyroidectomy remains the only curative treatment option for PHPT and preoperative vitamin D replacement may significantly reduce postoperative hypocalcemia rates.

The influence of preoperative vitamin D on the development of hypocalcemia occurrence requires more studies on postoperative hypocalcemia relationship with vitamin D after parathyroidectomy.

## References

[1] Machado NN, Wilhelm SM (2019). Diagnosis and Evaluation of Primary Hyperparathyroidism. Surg. Clin. North Am..

[2] Yeh MW (2013). Incidence and prevalence of primary hyperparathyroidism in a racially mixed population. J. Clin. Endocrinol. Metab.

[3] Syed H, Khan A (2017). Primary hyperparathyroidism: diagnosis and management in 2017. Polish Archives of Internal Medicine.

[4] Rejnmark L, Vestergaard P, Mosekilde L (2011). Nephrolithiasis and renal calcifications in primary hyperparathyroidism. J. Clin. Endocrinol. Metab..

[5] Bollerslev J (2019). Management of Endocrine Disease: Unmet therapeutic, educational and scientific needs in parathyroid disorders. Eur. J. Endocrinol.

[6] Yeh MW (2016). The Relationship of Parathyroidectomy and Bisphosphonates With Fracture Risk in Primary Hyperparathyroidism: An Observational Study. Ann. Intern. Med..

[7] Vestergaard P, Mosekilde L (2004). Parathyroid surgery is associated with a decreased risk of hip and upper arm fractures in primary hyperparathyroidism: a controlled cohort study. J. Intern. Med..

[8] Slominski AT, Kim T-K, Li W, Tuckey RC (2016). Classical and non-classical metabolic transformation of vitamin D in dermal fibroblasts. Exp. Dermatol..

[9] Slominski AT (2015). Detection of novel CYP11A1-derived secosteroids in the human epidermis and serum and pig adrenal gland. Sci Rep.

[10] Slominski AT (2012). In vivo evidence for a novel pathway of vitamin D_3_ metabolism initiated by P450scc and modified by CYP27B1. FASEB J..

[11] Bikle DD (2014). Vitamin D metabolism, mechanism of action, and clinical applications. Chem. Biol..

[12] Liu C (2020). A Practical Mathematic Method to Predict and Manage Hypocalcemia After Parathyroidectomy and Thyroidectomy. Ann. Otol. Rhinol. Laryngol..

[13] Gambardella C (2019). The role of prophylactic central compartment lymph node dissection in elderly patients with differentiated thyroid cancer: a multicentric study. BMC Surg.

[14] Jinih M, O’Connell E, O’Leary DP, Liew A, Redmond HP (2017). Focused Versus Bilateral Parathyroid Exploration for Primary Hyperparathyroidism: A Systematic Review and Meta-analysis. Ann. Surg. Oncol..

[15] Orr LE (2020). Skeletal effects of combined medical and surgical management of primary hyperparathyroidism. Surgery.

[16] Philips R (2019). Predicting transient hypocalcemia in patients with unplanned parathyroidectomy after thyroidectomy. Am J Otolaryngol.

[17] Lo CY, Tam SC (2001). Parathyroid autotransplantation during thyroidectomy: documentation of graft function. Arch Surg.

[18] El-Sharaky MI (2003). Assessment of parathyroid autotransplantation for preservation of parathyroid function after total thyroidectomy. Head Neck.

[19] Bergenfelz A, Lindblom P, Tibblin S, Westerdahl J (2002). Unilateral versus bilateral neck exploration for primary hyperparathyroidism: a prospective randomized controlled trial. Ann. Surg..

[20] Schneider DF, Mazeh H, Chen H, Sippel RS (2014). Predictors of recurrence in primary hyperparathyroidism: an analysis of 1386 cases. Ann. Surg..

[21] Yıldız, S. Y. *et al*. Tiroidektomi Sonrası Hipokalsemi Gelişiminde Paratiroid Hormon ve Diğer Faktörlerin Etkisi. **6**.

[22] Soares, C. S. P., Tagliarini, J. V. & Mazeto, G. M. F. S. Preoperative vitamin D level as a post-total thyroidectomy hypocalcemia predictor: a prospective study. *Braz J Otorhinolaryngol*, 10.1016/j.bjorl.2019.07.001 (2019) 10.1016/j.bjorl.2019.07.001 PMC9422554 31492617

[23] Falcone TE (2015). Correlating pre-operative vitamin D status with post-thyroidectomy hypocalcemia. Endocr Pract.

[24] Lang BH-H (2013). Does preoperative 25-hydroxyvitamin D status significantly affect the calcium kinetics after total thyroidectomy?. World J Surg.

[25] Al-Khatib T (2015). Severe vitamin D deficiency: a significant predictor of early hypocalcemia after total thyroidectomy. Otolaryngol Head Neck Surg.

